# *Toxoplasma gondii* non-archetypal strain induces lung inflammation during acute and early chronic infection in mice

**DOI:** 10.1017/S0031182025000538

**Published:** 2025-04

**Authors:** Alexandre Lazoski Bastilho, Ramayana M.M. Brito, José L. Andrade, José Bryan da Rocha Rihs, Isabela de Brito Duval, Marcelo Eduardo Cardozo, Ana Rafaela Antunes Porto, Luisa Braga do Amaral, Jorge Lucas Nascimento Souza, Geovanni Dantas Cassali, Brena K.C. Melo, Jully Anne B. Lemos, Rômulo S. Cavalcante, Lilian Bueno, Ricardo Toshio Fujiwara, Janeusa T. Souto, Luisa M.D. Magalhães, Valter Ferreira Andrade-Neto

**Affiliations:** 1 Department of Microbiology and Parasitology, Biosciences Center, Federal University of Rio Grande do Norte, Natal, Brazil; 2 Department of Parasitology, Institute of Biological Sciences, Federal University of Minas Gerais, Belo Horizonte, Brazil; 3 Department of Pathology, Institute of Biological Sciences, Federal University of Minas Gerais, UFMG, Belo Horizonte, Brazil; 4 Faculty of Health Sciences of the Trairí, Federal University of Rio Grande do Norte, Natal, Brazil

**Keywords:** atypical strains, lung inflammation, murine model, *Toxoplasma gondii*

## Abstract

*Toxoplasma gondii* non-archetypal strains have distinct virulence profiles and immunological activation in the host when compared with archetypal strains. The present work aims to perform an analysis of the inflammatory profile during acute and early chronic infection by *T. gondii* atypical strain in an experimental murine model. After euthanasia, blood was collected for the quantification of specific IgG antibodies and their subtypes (IgG1/IgG3) by ELISA; bronchoalveolar lavage (BAL) was realized and immunophenotyping of lymphocytes population was performed at 12- and 30-days post infection (dpi); the levels of IFN-γ, IL-12, IL-10, TNF-α, IL-6, IL-17, nitric oxide and total proteins were determined in the BAL supernatant. Tissue cyst burden was determined in the brain homogenate, and the parasite load in the lungs was assessed by quantitative reverse transcription polymerase chain reaction (qRT-PCR). Infection with the CK4 strain induced a lower brain cyst load similar parasite burden in the lungs, and higher levels of IgG1 and IgG3, when compared to ME49. The group infected with the CK4 strain presented higher levels of systemic IFN-γ, and both infected groups displayed similarly elevated levels of systemic TNF-α, IL-6 and IL-17 at 30 dpi, as well as higher numbers of CD4^+^ and CD8^+^ T lymphocytes in the acute stage of infection, followed by higher numbers of central and effector CD4^+^ T cells. IFN-γ levels in the BAL fluid were significantly higher in animals infected with the CK4 strain in both the acute and early chronic stage of infection, highlighting the involvement of the lung environment.

## Graphical abstract

**Figure:**
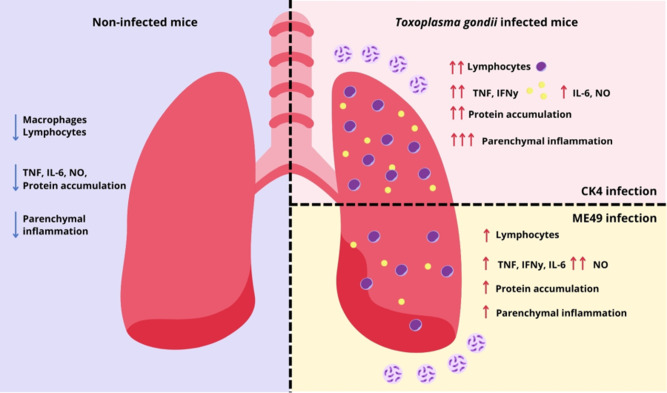

## Introduction

Toxoplasmosis is an infection caused by the intracellular protozoan *Toxoplasma gondii*, displaying high worldwide prevalence in both humans and animals (Hill et al., 2005; Furtado et al., 2011). The *T. gondii* population structure is marked by a great diversity of genotypes and strains. In the Northern Hemisphere, there is a predominance of 3 archetypal lineagess, referred to as types I, II and III according to specific allelic markers (Sibley and Ajioka, 2008; Su et al., 2012). A strain’s genetic signature can reflect its virulence profile, as type I strains can cause the death of mouse with just a single viable parasite, whereas types II and III strains are classified as of intermediate virulence and non-virulent, respectively, although the impact on the host also relies on the inoculum size and the host’s resistance and susceptibility profile to infection (Ajzenberg et al., 2004; Pena et al., 2008; Xiao and Yolken, 2015).

The genetic diversity of *T. gondii* worldwide is greater than initially thought, and genotypes distributed across South America were found to belong to a larger haplogroup, now identified as ‘non-archetypal’ or ‘atypical’ genotypes, that is distinct from the well-defined archetypal strains (Su et al., 2012). Atypical *T. gondii* strains display distinct virulence profile, as a result of single nucleotide polymorphisms (SNPs) in the coding sequences for specific proteins that are released by the parasite, and are crucial for triggering the host immune response, for the severity of infection (Brito et al., 2023a; Rico-Torres et al., 2024).

Primary *T. gondii* infection is usually asymptomatic, leads to latent infection. This latent infection may be reactivated in immunocompromised patients (Durieux et al., 2022). Pulmonary involvement was previously shown by Stajner et al. (2013), where a 15-year-old immunocompromised patient experiencing severe chest pain, with excess pleural fluid in the left lung, had a *T. gondii* atypical strain isolated from the bronchoalveolar lavage (BAL) fluid (BALF). In another study, an immunocompetent patient exhibited chest pain upon breathing, hepatosplenomegaly, fever and tachycardia, and was diagnosed with acute *T. gondii* infection (Leal et al., 2007); later on, this strain was isolated and characterized as a highly virulent atypical one (Pena et al., 2021). Furthermore, the association of *T. gondii* with lung diseases have also been explored, with patients with lung cancer displaying the highest *T. gondii* seroprevalence (Cong et al., 2015; Bajnok et al., 2019). The high seroprevalence of *T. gondii* infection among patients with lung diseases highlights its potential role in exacerbating respiratory conditions (Li et al., 2020).

This evidence raises questions regarding the impact *T. gondii* infection on the lung environment and how atypical strains might contribute to the lung pathology by triggering local and systemic inflammation. In this context, we evaluated the ability of a *T. gondii* atypical strain to stimulate inflammatory responses and tissue damage in the lungs using murine models of acute and early chronic infection.

## Materials and methods

### Parasite maintenance

In this study, 2 strains of *T. gondii* were used: the clonal ME49 strain (Type II strain) and an atypical strain TgCKBrRN4 (CK4; ToxoDB #163), previously isolated by Clementino-Andrade et al. (2013). The strains were obtained from the Laboratory of Toxoplasmosis, at the Federal University of Minas Gerais and maintained at the Laboratory of Malaria and Toxoplasmosis Biology (LABMAT), in the Department of Microbiology and Parasitology (DMP) at Federal University of Rio Grande do Norte (UFRN) through passages in Swiss mice.

### Experimental protocol

Female Swiss mice aged between 7 and 8 weeks were divided into 3 groups, consisting of 10 animals each: the group infected with the clonal strain (ME49); the group infected with the atypical strain (CK4) and the control group of not infected mice (Ni). The animals were orally infected with 10 tissue cysts and kept infected during 12 days for assessing the acute infection, and 30 days for the early chronic infection. During this period, the signs of acute infection were monitored and recorded, along with the post-infection survival rate. The sickness score, used to evaluate the disease progression, was calculated based on the clinical signs displayed by infected mice as piloerection (PE), development of ascites (DA), weight loss (WL); shivering (S), trunk curl (TC), lethargy (LE), dehydration (D) and paralysis (PA), using the formula PE + DA + WL + S + TC + LE + 3*(D + PA), according to the analysis previously published by Vandermosten et al. (2018).

### Euthanasia and blood collection

On the 12^th^ (acute stage) and 30^th^ (chronic stage) dpi, the animals were anesthetized using a combination of dissociative anesthetic (ketamine) combined with alpha-2 adrenoceptor agonists (xylazine) administered intraperitoneally. Then, their thoracic cavities were opened to expose the heart; blood samples were collected by cardiac puncture and the blood was kept at room temperature (RT) for approximately 30 min to allow clot retraction and serum separation. Serum samples were obtained after centrifugation at 1800 rpm for 10 min at RT and subsequently stored at −20 °C until antibody and cytokines level determination by ELISA.

### Bronchoalveolar lavage fluid

BALF was collected to examine the cellular populations in the lower respiratory tract. This procedure involved making an incision in the upper part of the trachea to insert a catheter (Safety Catheter® 18 G × 1¾″) connected to a 3 mL syringe. Subsequently, 3 intratracheal washes with 1 mL of PBS at 4 °C were performed. After collection, BALF was centrifuged at 300 *g* for 10 min at 4 °C. The supernatant was separated and stored at −80 °C for measurement of total proteins, nitric oxide and cytokines, as described below. The pellet was re-suspended in 1 mL of 1× phosphate buffered saline (PBS) and the total number of cells was determined by counting in a Neubauer chamber. The obtained cells were kept in ice bath until phenotyping. After BALF collection, samples of the lung were harvested for histopathological analysis and for quantification of the parasite load.

### Tissue cyst count

After perfusion, the brains of mice infected with either parasite strain were removed on 30 dpi to quantify the tissue cyst burden. For this purpose, each brain was individually macerated in 1 mL of 1× PBS until complete tissue dissociation. Once properly homogenized, 2 aliquots of 25 µL of the brain tissue homogenates were placed on microscopic slides, and the cyst burdens were counted under an optical microscope (at 100× magnification). The data were presented as the average number of cysts per mL of brain homogenate.

### DNA extraction and parasite load quantification by *qRT-PCR*

Lung tissues were collected at 12 and 30 dpi and stored at −80 °C for later analysis. The DNA extraction was performed according to Pitcher et al. (1989) using 30 mg of tissue. Samples were homogenized (TissueLyser LT – Qiagen, Hilden, Germany) in 100 μL of TE buffer (Tris-HCl 1 M; EDTA 0·5 M; pH 8·0). The concentration and purity of extracted DNA were measured using NanoDrop 2000 Spectrophotometer (Thermo Fisher Scientific Inc., USA).

The qPCR was performed using a total volume of 10 μL containing 5 μL of PowerUP™ Sybr green master mix kit (Applied Biosystems™), 0·3 μL of each primer (10 μM) Rep-529 F (5ʹ-AGAGACACCGGAATGCGATCT-3ʹ) and Rep-529 R (5ʹ-TTCGTCCAAGCCTCCGAC-3ʹ) and 3·4 μL of ultrapure nuclease-free water. Thermal cycling was performed using a 7500 Real-Time PCR system (Applied Biosystems™), starting with an initial step at 95 °C for 10 min followed by 40 cycles of denaturation at 95 °C for 15 s and annealing/extension at 60 °C for 60 s. Melting curve analysis was performed from 70 °C to 95 °C at 0·1 °C/s. The quantification of the samples was determined using the standard curve method for absolute quantification. The standard curves were prepared from serial dilutions of DNA extracted from 10⁷ tachyzoites of *T. gondii*. The results were expressed as the number of parasites per milliliter (mL) of extracted tissue DNA.

## Quntification of anti-*T. gondii* total IgG and IgG1/IgG3

The serum concentration of specific anti-*T. gondii* IgG antibodies, as well as their subclasses IgG1 and IgG3, was determined using an in-house enzyme-linked immunosorbent assay (ELISA), as previously described, with minor alterations (Oliveira et al., 2016; Clementino-Andrade et al., 2020; Brito et al., 2023b). Briefly, 96-well plates were coated with 1 μg/mL per well in a volume of 100 μL of *T. gondii* antigen solution for 2 h at 37 °C. After coating, the plate was washed 4 times with 0·05% PBS-Tween-20 washing solution.

The blocking step was performed using 200 μL/well of 3% BSA solution, with incubation for 1 h at 37 °C, protected from light. After incubation, the blocking solution was removed, and 50 μL/well of the diluted serum samples (1:100) were added in duplicate. The plate was incubated for 1 h at 37 °C, protected from light. After washing, 100 μL/well of the respective secondary antibodies conjugated to peroxidase, anti-IgG (1:30 000), anti-IgG1 (1:10 000) and anti-IgG3 (1:10 000) (Sigma–Aldrich, Saint Louis, USA) were added. The plate was incubated for 1 h under the same conditions described above. After incubation, another round of washing was performed. Following the final wash, 50 μL/well of the o-phenylenediamine dihydrochloride (OPD) substrate solution was added, and the plate was incubated for 20 min at RT, protected from light. After this period, the reaction was stopped by adding 30 μL per well of stop solution (2 M H_2_SO_4_). The plate was read using a spectrophotometer at a wavelength of 490 nm.

The results were calculated based on the cutoff point, which was determined by the mean absorbance of 6 known negative serum samples plus 3 times their standard deviation. Sera with reactivity index (RI) equal to or greater than 1 were considered positive, while values below 1 were considered negative. The levels of specific antibodies were presented according to the identified optical density (OD).

### Quantification of inflammatory cytokines in the BALF and sera

The quantification of the cytokines IFN-γ, TNF-α, IL-17, IL-6, IL-10 and IL-12 in the BALF supernatant and serum of mice was performed using the ELISA Ready-SET-GO!® kits (eBioscience™, USA). The technique applied is based on the principle of sandwich ELISA method associated with a standard curve, following the manufacturer’s instructions for each kit.

### Determination of nitric oxide and protein levels in the BALF

The levels of nitrite in the BALF were determined using the Griess colorimetric assay, an indirect method for detecting the presence of nitric oxide in biological samples. In summary, 100 μL/well of the BALF supernatant were added to a 96-well plate, in duplicate. After adding the samples, 100 μL/well of the Griess reagent (1% sulfanilamide in 5% H_2_PO_4_ and 0·1% N-1-naphthylethylenediamine in distilled water) were added. After adding the reagent, the plate was incubated for 10 min at RT and protected from light. The absorbance was measured using a spectrophotometer at a wavelength of 540 nm. The concentration of nitrite in the samples was determined by interpolation from the standard curve.

The total protein accumulation in the BALF was determined using Pierce^TM^ BCA assay kit (ThermoFisher Scientific, USA) according to the manufacturer’s instructions.

### Histopathological analysis of the lungs

The mouse lungs were removed and fixed by immersion in 10% buffered paraformaldehyde (PFA). After 48 h, they were removed from formalin and transferred to 70% ethanol. Subsequently, the lungs were longitudinally sectioned and subjected to dehydration and impregnation with paraffin. The tissues were then embedded in paraffin blocks, and 4 µm-thick sections were cut using a microtome. The sections were stained with hematoxylin and eosin. After staining, the sections were mounted on slides and covered with coverslips using synthetic resin (Entellan-Merck) for subsequent evaluation by light microscopy.

The lung tissue alterations were evaluated using a semi-quantitative scoring system, as described by Gori et al. (2019), with minor modifications. Scores ranging from 0 to 4 were assigned based on the presence of alterations in alveolar thickness and structure, occurrence of hemorrhage, vascular congestion and presence of inflammatory infiltrate. The scores were assigned after analysing approximately 20 fields across the entire extent of the histological sections. The scores were assigned independently for each of the 7 animals in each group. Finally, the mean scores for the groups were graphically represented. The analyses were conducted in a blinded manner.

### Multiparametric flow cytometry

Multiparametric flow cytometry was performed to analyse the main lymphocyte populations during both the acute and chronic stages of *T. gondii* infection, following a previously published study protocol (Vieira-Santos et al., 2024). Cell staining began with the addition of the viability dye (LiveDead, AF700, BD Bioscience, San Jose, CA), followed by a 10-min incubation at 4 °C. After incubation, cells were washed twice by centrifugation at 300 ×*g* for 8 min at 4 °C with 1× PBS. Next, cells were incubated with 50 μL of a surface antibody mix containing 10% serum from control mice for 15 min at 4 °C. The antibodies used included CD44 BV510, CD3 BV570, CD19 FITC, CD4 PerCP, CD62L PE-CF, CD8 APC, LD AF700 and CD69 APC-Cy7 (BD Bioscience, San Jose, CA). Cells were then washed twice more with 1× PBS as previously described and fixed with 2% PFA for 20 min at RT. After fixation, cells were washed twice with 1× PBS and transferred to FACS tubes. Data acquisition was performed on an LSR Fortessa (BD Biosciences, USA) and analyzed using FlowJo software (Tree Star, Ashlan, OR, USA).

### Statistical analysis

The normality of the data presented here was assessed using the Shapiro–Wilk test. Parametric data were analysed using one-way ANOVA followed by Tukey’s post-hoc test, or Student’s *t*-test when applicable. Non‐parametric data were analysed using the Kruskal–Wallis test followed by Dunn’s post‐test, or Mann–Whitney test when applicable. Pearson’s correlation coefficient was used for correlation analysis. Principal Component Analysis was performed using the web tool ClustVis (Metsalu and Vilo, 2015) for visualization of clusters. The data were expressed as mean ± standard deviation, and statistical significance was considered when *P* < 0·05. The data presented here is representative of 3 independent experiments.

## Results

### Evolution of the infection by an non-archetypal strain of *T. gondii*

**Figure 1.**
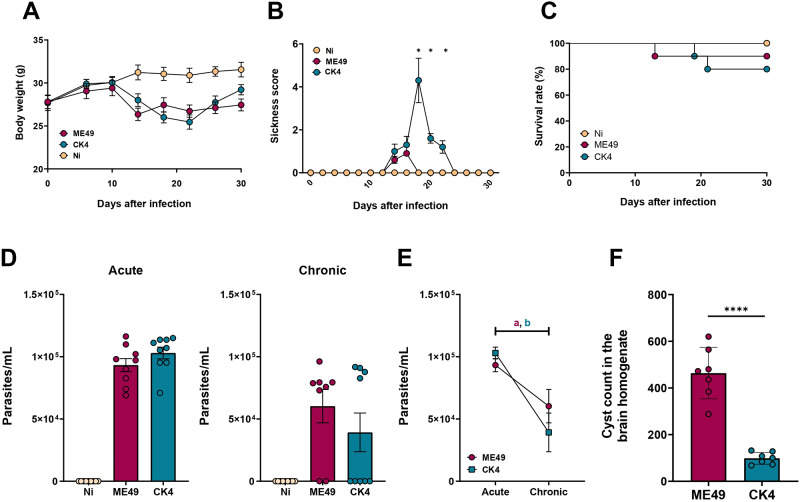
Progression of *T. gondii* infection. Swiss mice were orally infected with 10 cysts of *T. gondii* ME49 or CK4 strain and monitored for 30 days. (A) Monitoring of body weight loss during the course of infection. (B) Sickness score based on the clinical signs presented by infected mice during the infection course. (C) Survival rate of infected mice, compared to non-infected (Ni) mice (10 animals per group). (D) Parasite load quantified by qRT-PCR in the lungs of mice during acute (left) and chronic (right) infection stages. (E) Comparison in the parasite load on the lungs of ME49- and CK4-infected mice during acute and chronic infection (a = statistical difference between acute and chronic stage of infection by ME49, *P* = 0·006; b = statistical difference between acute and chronic stage of infection by CK4, *P* = 0·001). (F) Average number of tissue cyst in the brain homogenate of Swiss mice infected with ME49 or CK4. The data are presented in bar graphs with overlapping dots representing the sample distribution, and maximum and minimum values. Survival rate was analysed using Kaplan–Meier estimate. The results presented here are representative of 3 independent experiments. *P < 0·05; ****P < 0·0001.

During the first 10 dpi, typical clinical signs of the acute infection were observed in both infected groups, such as weight loss and piloerection. As the infection progressed, the infected groups began to lose weight from the d14 pi (ME49 vs Ni: *P* < 0·0001; CK4 vs Ni *P* *=* 0·007) (Figure 1A). Animals infected with either ME49 or CK4 strain showed marked reduction in body weight, when compared to non-infected animals starting at 14th dpi up to 22nd dpi (*P* < 0·0001); there was a regain of weight from the 26th dpi in the group infected with CK4, reaching to a weight recovery on the 30th dpi with no significant differences from the control group. At this time point, the group infected with ME49 remained with a significant reduction in body weight when compared to non-infected control animals (*P* = 0·0005). The group infected with CK4 strain showed development of ascites in 60% of the animals, and this group presented with a higher sickness score (*P* < 0·05) when compared to ME49-infected mice (Figure 1B). Throughout the experiment, a low mortality rate was observed in both groups. The survival rate of individuals infected with the clonal strain ME49 was 90%, while those infected with CK4 was 80% (Figure 1C).

The parasite load in the lungs of both ME49- and CK4-infected mice was determined by RT-qPCR during both acute and chronic stages. Similar lung tissue parasite burdens were detected in both infected groups of animals, with no significant differences between the 2 strains at either time point analysed (Figure 1D). However, the parasite load significantly decreased from acute to chronic stage (ME49: *P* = 0·006; CK4: *P* = 0·001) (Figure 1E). When comparing the tissue cyst burden in the brains of infected mice at 30 dpi, it was found that infection with the ME49 strain led to significantly higher number of brain cysts in mice compared to CK4 (*P* < 0·0001) (Figure 1F).

**Figure 2.**
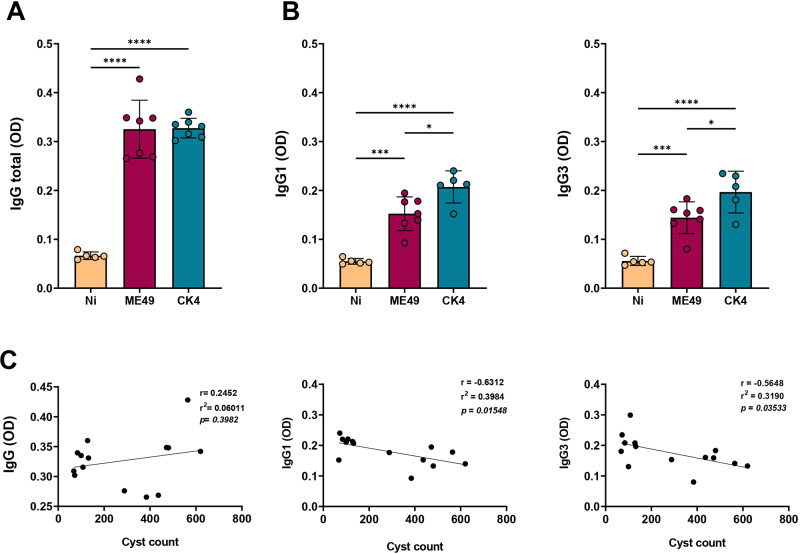
Assessment of anti-*T. gondii* IgG antibodies. (A) Measurement of specific anti-*T. gondii* IgG total (first), and its subtypes IgG1 (B, left) and IgG3 (B, right). (C) Pearson’s correlation of the tissue cyst burden and the levels of specific antibodies. The data are presented in bar graphs with overlapping dots representing the sample distribution, and maximum and minimum values. The results presented here are representative of 3 independent experiments. *P < 0·05; ***P < 0·001; ****P < 0·0001.

At 30 dpi, the profile of humoral immune response was evaluated, and it was observed that both strains were able to stimulate the production of specific IgG antibodies when compared to non-infected mice (*P* < 0·0001), with no significant differences between the infected groups (Figure 2A). However, when analysing the levels of IgG subtypes IgG1 and IgG3, infection with the atypical strain CK4 induced significantly higher levels of these antibodies when compared to ME49 strain (IgG1: *P* = 0·015; IgG3: *P* = 0·003) (Figure 2B, left and right). In order to find a relationship between the antibody levels and the brain cyst burden, correlation analyses were performed, revealing an inverse relationship between cyst burden and levels of IgG1 (Figure 2C, middle) and IgG3 (Figure 2C, right) produced during *T. gondii* infection.

### Sytemic inflammation during early *T. gondii* chronic infection

**Figure 3.**
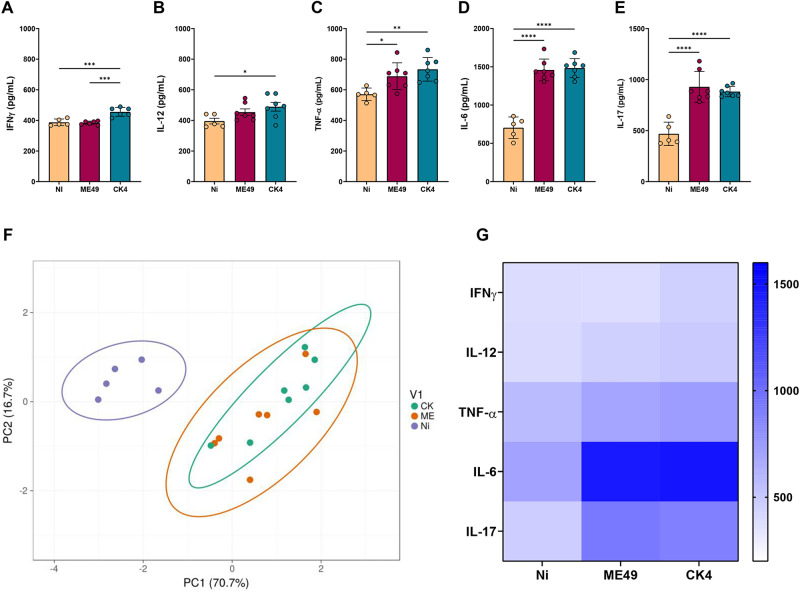
Systemic inflammatory cytokines during *T. gondii* infection by clonal and atypical strains. Levels of the inflammatory cytokines IFN-γ (A), IL-12 (B), TNF-α (C), IL-6 (D) and IL-17 (E) quantified in the sera of non-infected (Ni), ME49- and CK4-infected Swiss mice at 30 dpi. (F) Principal Component Analysis (PCA) of the 3 groups analysed, based on the levels of systemic cytokines. (G) Heatmap highlighting the pattern of inflammatory cytokines within the groups. The data are presented in bar graphs with overlapping dots representing the sample distribution, and maximum and minimum values. The results presented here are representative of 3 independent experiments. *P < 0·05; **P < 0·01; ***P < 0·001; ****P < 0·0001.

The systemic inflammatory profile was determined by the quantification of the inflammatory cytokines IFN-γ, IL-12, TNF-α, IL-6 and IL-17 in the serum of *T. gondii* infected mice at 30 dpi. The group infected with the CK4 strain displayed increased levels of systemic IFN-γ (Figure 3A), when compared to the non-infected (*P* = 0·0004) and ME49-infected (*P* = 0·0002) groups. Additionally, CK4-infected mice displayed higher levels (*P* = 0·04) of IL-12 when compared to the non-infected group (Figure 3B). Both ME49- and CK4-infected groups had higher and similar levels of TNF-α (*P* = 0·03; *P* = 0·004, respectively) (Figure 3C), IL-6 (*P* < 0·0001) (Figure 3D) and IL-17 (*P* < 0·0001) (Figure 3E) when compared to the non-infected group.

Principal component analysis was performed and showed that both ME49- and CK4-infected groups represent similar clusters and can be considered completely distinct from the non-infected group, based on the level of systemic inflammation (Figure 3F), as evidenced by the differences in the levels of the analysed inflammatory cytokines (Figure 3G).

### Pulmonary inflammation during infection by *T. gondii* non-archetypal strain

**Figure 4.**
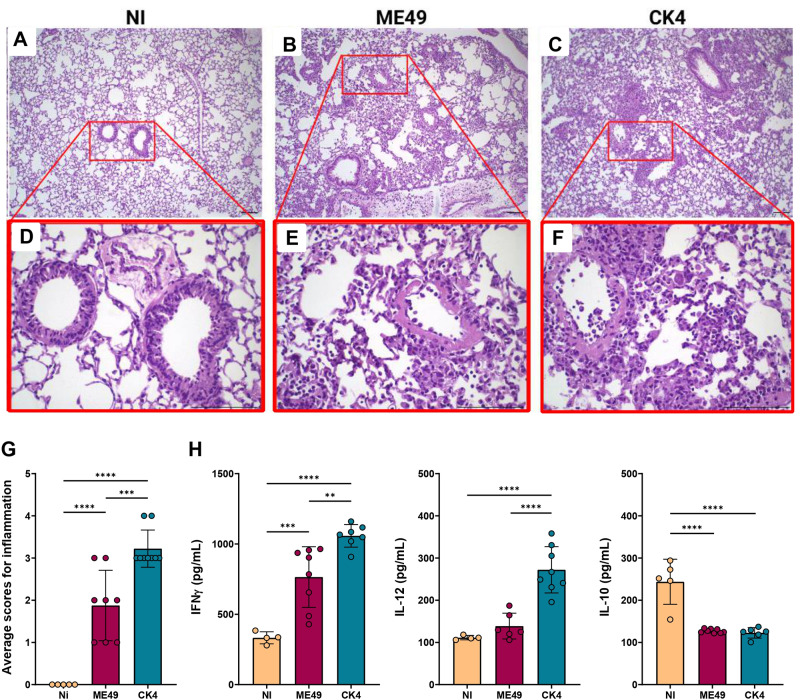
Histopathological analyses and cytokine profile in the lungs during acute infection by *T. gondii*. (A) Representative image of the lung histopathology during acute infection by either ME49 and CK4 strains. Figures A, B and C: 10× magnification; Figures D, E and F: 40× magnification. (G) Level of inflammation in lung tissue of infected groups compared to the control group (Ni), showing a higher inflammatory process in the lungs of mice infected with the atypical strain CK4. (H) Levels of the cytokines IFN-γ, IL-12 and IL-10 quantified in the BALF of non-infected, ME49- and CK4-infected Swiss mice at 12 dpi. The data are presented in bar graphs with overlapping dots representing the sample distribution, and maximum and minimum values. The results presented here are representative of 3 independent experiments. **P < 0·01; ***P < 0·001; ****P < 0·0001.

Analysis of the inflammatory patterns in the lungs of *T. gondii-*infected mice during acute infection showed that mice infected with ME49 exhibited a discrete inflammatory infiltrate, composed mainly of mononuclear cells, presence of hyperemia and thickening of vascular wall (Figure 4B, E). The lungs of mice infected with the CK4 strain, exhibited heavier inflammation, with extensive impairment of lung parenchyma in addition to severe alveolar thickening, and an extensive and diffuse inflammatory infiltrate, also composed mainly of mononuclear cells (Figure 4C, F). Overall, acute infection by the CK4 strain induced a higher level of inflammation in the lung tissue (Figure 4G), when compared to the non-infected (*P* < 0·0001) and ME49-infected (*P* = 0·0004) groups.

To assess the impact of acute infection on the production of cytokines, the levels of IFN-γ, IL-12 and IL-10 were quantified in the BALF of ME49- and CK4-infected mice at 12 dpi. Infection by both strains was able to induce increased levels of IFN-γ as compared to control mice (ME49: *P* = 0·0008; CK4: *P* < 0·0001), with CK4 infection inducing higher levels compared to both control and ME49-infected (*P* = 0·005) groups (Figure 4H, left). The atypical strain was also able to induce high levels of IL-12 (*P* < 0·0001) (Figure 4H, middle), and the levels of IL-10 were found to be decreased in both infected groups, when compared to the non-infected group (*P* < 0·0001) (Figure 4H, right).

**Figure 5.**
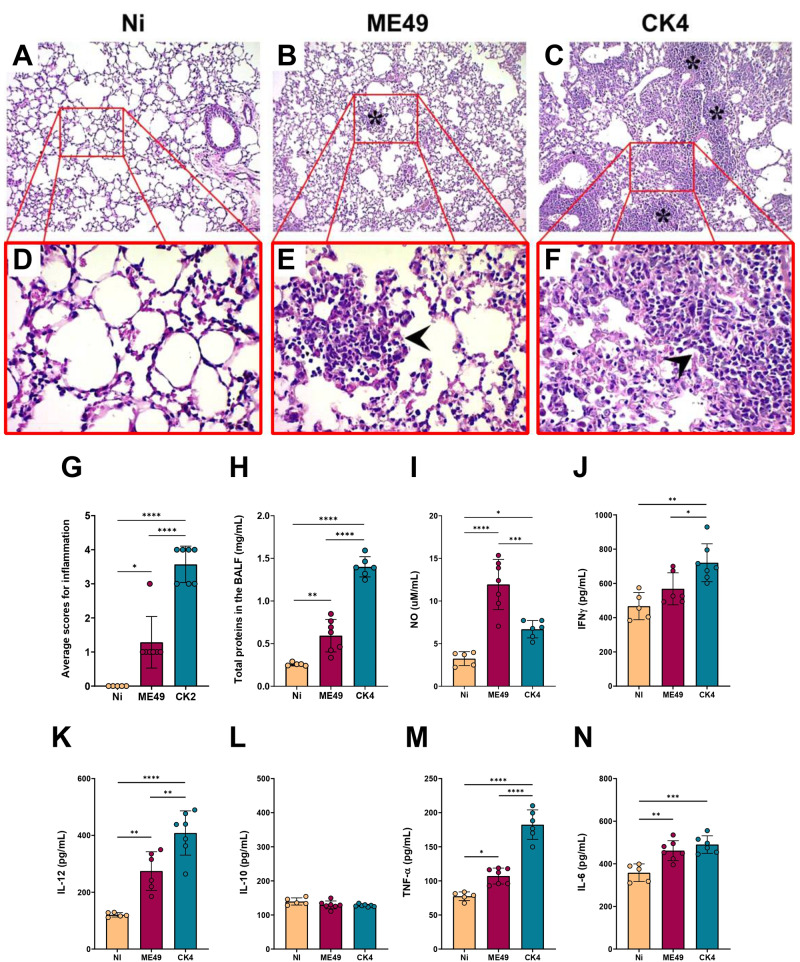
Histopathological analyses and cytokine profile in the lungs during chronic infection by *T. gondii*. Figures A, B and C: 10× magnification; Figures D, E and F: 40× magnification. Lung parenchyma was analysed, and asterisks represent areas containing inflammatory infiltrate, while arrowheads indicate the mononuclear pattern of inflammatory cells. (G) Level of inflammation in lung tissue of infected groups compared to the control group (Ni), showing a higher inflammatory process in the lungs of mice infected with the atypical strain CK4. (H) Quantification of total proteins in the BALF of non-infected and infected groups. (I) Levels of nitric oxide (NO) determined in the BALF of non-infected and infected groups. Levels of the cytokines IFN-γ (J), IL-12 (K), IL-10 (L), TNF-α (M) and IL-6 (N) quantified in the BALF of non-infected, ME49- and CK4-infected Swiss mice at 30 dpi. The data are presented in bar graphs with overlapping dots representing the sample distribution, and maximum and minimum values. The results presented here are representative of 3 independent experiments. *P < 0·05; **P < 0·01; ***P < 0·001; ****P < 0·0001.

During the chronic stage of infection, histopathological evaluation of the lungs revealed, in addition to the disorganization of the lung parenchymal architecture, the presence of a strong inflammatory infiltrate, especially in the mice infected with CK4 (Figure 5C and F). The group infected with ME49 also showed elevated levels of inflammatory infiltrate (Figure 4B and E) compared to the non-infected group. Infection with the *T. gondii* CK4 strain was capable of causing greater disorganization of the lung architecture and greater tissue inflammation than ME49 infection (*P* < 0·0001) (Figure 5G). Furthermore, the quantification of total proteins in the BALF supernatant showed that mice infected with the atypical *T. gondii* CK4 strain had a higher concentration of total proteins at 30 dpi (*P* < 0·0001) (Figure 5H), and the levels of nitric oxide in the BALF were higher in infected mice compared to the control group (ME49: *P* < 0·0001; CK4: *P* = 0·03). However, the atypical strain induced significantly lower levels of nitric oxide compared to the ME49-infected group (*P* = 0·0007) (Figure 5I).

Regarding the cytokine levels in the lungs during chronic infection by either ME49 or CK4 strains, it was found that the atypical strain was able to induce higher levels of IFN-γ, when compared to the non-infected group (*P* = 0·001) and the group infected with ME49 (*P* = 0·03) (Figure 5J). While the levels of IL-10 did not show any significant differences between the groups (Figure 5L), the levels of IL-12 (Figure 5K) and TNF-α (Figure 5M) were significantly increased in the BALF of mice infected with the CK4 strain (CK4 vs Ni: *P* < 0·0001; CK4 vs ME49: *P* = 0·004). Both ME49 and CK4 infection lead to similarly increased levels of IL-6 in early chronicity, when compared to the non-infected animals (ME49 *P* = 0·002; CK4 *P* = 0·0004; Figure 5N).

### Immunophenotyping of lymphocyte populations in the BALF

In order to analyse the lymphocyte populations, infiltrated in the lungs of mice infected by *T. gondii*, multiparametric flow cytometry (Sup. Figure 1) was performed during acute and chronic stages of infection.

**Figure 6.**
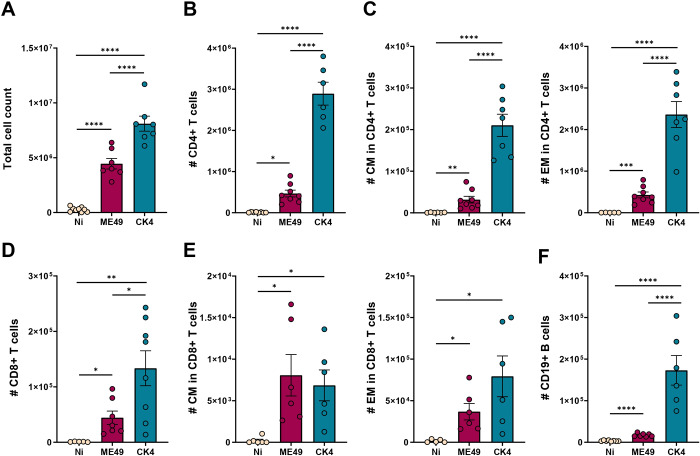
Immunophenotyping of lymphocytes population in the lungs during acute infection by *T. gondii*. (A) total cell count in the BALF of non-infected, ME49- and CK4-infected groups during the acute stage of infection, at 12 dpi. (B) Number of Live/CD3^+^CD4^+^ T lymphocytes. (C) Number of Live/CD3^+^CD4^+^CD44^+^CD62L^+^ (CM, left), and number of Live/CD3^+^CD4^+^CD44^+^CD62L^−^ (EM, right) T lymphocytes. (D) Number of Live/CD3^+^CD8^+^ T lymphocytes. (E) Number of Live/CD3^+^CD8^+^CD44^+^CD62L^+^ (CM, left), and number of Live/CD3^+^CD8^+^CD44^+^CD62L^−^ (EM, right) T lymphocytes. (F) Number of Live/CD3^–^CD19^+^ B lymphocytes. The data are presented in bar graphs with overlapping dots representing the sample distribution, and maximum and minimum values. The results presented here are representative of 3 independent experiments. *P < 0·05; **P < 0·01; ***P < 0·001; ****P < 0·0001.

During acute infection, a higher number of cells was recovered from the BALF from both infected groups, and the group infected with the CK4 strain exhibited significantly higher number of cells when compared to the non-infected (*P* < 0·0001) and ME49-infected groups (*P* < 0·0001) (Figure 6A). Within the infiltrated lymphocytes, the group infected with the CK4 strain displayed increased numbers of CD4^+^ T lymphocytes (*P* < 0·0001) (Figure 6B), with higher numbers of central memory (CM) (*P* < 0·0001) (Figure 6C, left) and effector memory (EM) CD4^+^ T cells (*P* < 0·0001) (Figure 6C, right) when compared to both non-infected and ME49-infected groups. Regarding the population of CD8^+^ T lymphocytes, higher numbers were found in mice infected with the atypical strain (Figure 6D), when compared to the non-infected (*P* = 0·003), and ME49-infected (*P* = 0·02) groups. Although the CM and EM CD8^+^ T lymphocytes were higher than the non-infected group (ME49: *P* = 0·02; CK4: *P* = 0·03) (Figure 6E), no differences were found between ME49- and CK4-infected groups. The population of B cells was also found to be significantly increased in mice infected with the atypical CK4 strain (*P* < 0·0001) (Figure 6F).

**Figure 7.**
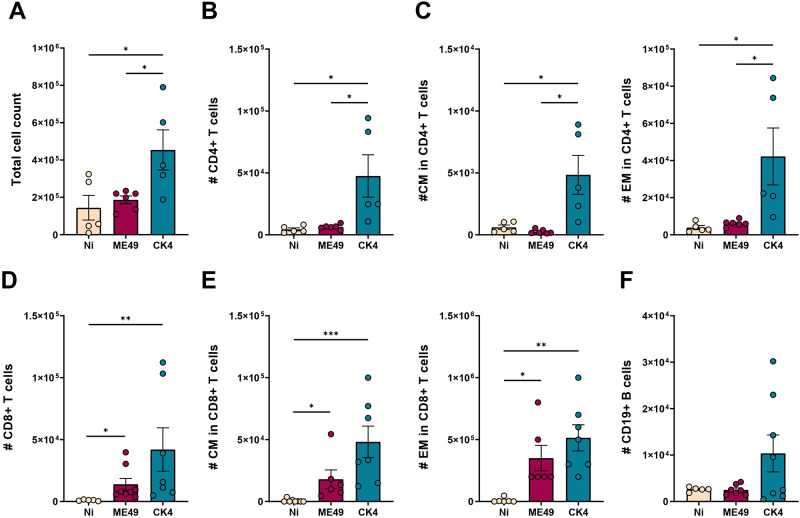
Immunophenotyping of lymphocytes population in the lungs during chronic infection by *T. gondii*. (A) Total cell count in the BALF of non-infected, ME49- and CK4-infected groups during the chronic stage of infection, at 30 dpi. (B) Number of Live/CD3^+^CD4^+^ T lymphocytes. (C) Number of Live/CD3^+^CD4^+^CD44^+^CD62L^+^ (CM, left), and number of Live/CD3^+^CD4^+^CD44^+^CD62L^−^ (EM, right) T lymphocytes. (D) Number of Live/CD3^+^CD8^+^ T lymphocytes. (E) Number of Live/CD3^+^CD8^+^CD44^+^CD62L^+^ (CM, left) and number of Live/CD3^+^CD8^+^CD44^+^CD62L^−^ (EM, right) T lymphocytes. (F) Number of Live/CD3^-^CD19^+^ B lymphocytes. The data are presented in bar graphs with overlapping dots representing the sample distribution, and maximum and minimum values. The results presented here are representative of 3 independent experiments. *P < 0·05; **P < 0·01; ***P < 0·001.

To determine whether the lung lymphocyte patterns observed during acute infection persisted in the chronic stage, immune cell populations were analysed in the BALF of infected mice. During chronic infection, the CK4-infected group continued to show elevated total cell counts compared to the non-infected (*P* = 0·02) and ME49-infected (*P* = 0·03) groups (Figure 7A). Notably, CD4^+^ T lymphocytes remained significantly elevated in the group infected with the atypical strain (*P* = 0·02), with CK4 inducing a higher cell count than the ME49-infected group (*P* = 0·02) (Figure 7B). Within CD4^+^ T cells, both CM and EM subsets (Figure 7C, left and right) were significantly higher in CK4-infected mice than in the non-infected group (*P* = 0·009), and also than in ME49-infected mice (*P* = 0·005).

Similarly, the CD8^+^ T lymphocyte population continued to exhibit increased numbers in both infected groups during chronic infection (ME49: *P* = 0·03; CK4: *P* = 0·002) (Figure 7D). As with CD4^+^ T cells, both CM and EM subsets of CD8^+^ T cells showed sustained higher cell numbers compared to the non-infected animals (ME49: *P* = 0·04; CK4: *P* = 0·0003) (Figure 7E). Finally, during chronic infection, no differences were found in the number of CD19^+^ B lymphocytes between the 3 analysed groups (Figure 7F).

These findings indicate that the elevated lymphocyte profile, observed during acute infection, continues into the chronic stage. Infection with the atypical strain used here induced an enhanced immune response as compared to infection with a typical type II strain both in the acute and in the early chronic stage of infection.

## Discussion

*Toxoplasma gondii* atypical strains display unique genetic characteristics that may imply distinct activation of the immune response and the pathology triggered in the infected host (Brito et al., 2023a). In this work, a *T. gondii* atypical strain (TgCkBrRN4) was compared to a classical type-II strain (ME49), evaluating the infection progression and the development of lung inflammation in experimentally infected mice.

To evaluate the course of infection, mice were monitored during 30 dpi. Among the mice infected with the atypical strain CK4, a greater reduction in body weight was observed, which may be attributed to inflammatory activation during this period of infection, which can be associated with greater intestinal impairment (Hatter et al., 2018). In addition to the weight loss, occurrence of ascites was also registered. Ascites is primarily associated with functional difficulties in the body’s circulatory system, leading to sodium and water accumulation, particularly in the abdominal region, and is present in cases of renal and hepatic infection (Cárdenas et al., 2000). It is known that *T. gondii* infection can cause hepatosplenomegaly and subsequently ascites due to the dysfunctions induced (Blaakaer, 1986). In the study presented here, ascites was found only in the CK4-infected. This may suggest the ability of this atypical strain to contribute to peritonitis, as it was shown that mice infected with a virulent strain of *T. gondii* displayed marked ascites with increased number of neutrophils and nitric oxide in the peritoneum fluid (Bottari et al., 2014).

Studies on murine infection with atypical strains of *T. gondii* have shown that tissue cysts formed during infection with atypical strains can exhibit tropism for different organs, in addition to the brain, such as the liver, lungs and heart (Bottari et al., 2014; Fernández-Escobar et al., 2021). Furthermore, the virulence profile and the mechanisms involved in immune activation can influence the parasite’s dissemination within the host (Betancourt et al., 2019). Atypical genotype strains exhibit distinct virulence profiles in murine experimental models, raising the discussion about the behaviour of the parasite and its relationship with the host (Clementino-Andrade et al., 2013; Fernández-Escobar et al., 2021). In the present study, a significant difference in the brain cyst burden was found between animals infected with ME49 and CK4; animals infected with ME49 had more tissue cysts compared to the group infected with the atypical CK4 strain. High parasite load was found in the lungs of infected mice during both acute and chronic infection, with the burden decreasing as the infection reached the chronic stage.

Since atypical strains result from genetic recombination, it is possible to hypothesize that CK4 could have a longer cycle of tachyzoite migration, or it could also be related to differential expression of transcription factors, such as AP2IV-4, which regulate the establishment of chronic infection (Radke et al., 2018). On the other hand, it is known that, although there is a tropism for certain body regions such as muscles and the brain, tissue cysts can also occur in other organs depending on the strain analysed (Dubey et al., 1998). An interesting fact, is that in a previous work analysing the impact of another atypical *T. gondii* strain, TgCKBrRN2 (CK2), belonging to the same genotype (Clementino-Andrade et al., 2013), CK2 strain displayed distinct behaviour in the development of brain cysts and contributed to significant changes in the microglia population (Brito et al., 2023b). Taking this into consideration, further studies are needed to better understand the pathways triggered during infection that might contribute to higher or lower brain cyst burdens, in addition to the lungs colonization by the parasite during both acute and chronic infection. Besides, CK4 strain may also have tropism for other body locations, requiring a comprehensive evaluation of distinct organs at different time points of infection to further understand its dissemination and encystment patterns.

Analysis of total IgG antibodies and their subclasses evaluated in this study showed they were elevated in the infected groups, indicating an adequate humoral immunologic response to *T. gondii* infection. Regarding the subtypes evaluated here, IgG3 represents the main subtype produced in response to infection by intracellular parasites (Tebo et al., 2001) and that IgG1 also plays an important role during *T. gondii* infection, with studies showing that IgG1 is induced during *T. gondii* infection by IFN-γ (Correa et al., 2007; Sana et al., 2022). This fact can be supported by the high levels of IFN-γ found during acute and chronic stages of infection, as presented here, shedding light on the parasite dynamics and its relationship with the immune activation.

Herein, the levels of systemic inflammatory cytokines were evaluated. During chronic infection, high levels of systemic IFN-γ were found in mice infected with the atypical strain, and no differences in the systemic levels of TNF-α, IL-6 and IL-17 between ME49 and CK4 infected mice were found, indicating consistency in the inflammatory process and the host’s immune response to the parasite. However, distinct patterns of inflammatory cytokine production can be found when comparing the response to infection with different *T. gondii* strains in mice (Costa et al., 2020). The ability of *T. gondii* to induce elevated levels of systemic IL-17 is noteworthy, as this cytokine is a critical inflammatory mediator, and its importance in defence against intracellular parasites has been reported (Vesely et al., 2020; Mills, 2023). Indeed, studies have highlighted the role of IL-17 during protozoan infections such as *Trypanosoma cruzi* infection (Santos et al., 2009; Guedes et al., 2010, 2012), and *Leishmania* infection (Banerjee et al., 2016; Dietze-Schwonberg et al., 2019). In the context of *T. gondii* infection, Th17 axis was shown to be crucial in controlling infection and mortality in mice, as IL-17A-deficient C57BL/6 mice displayed higher mortality followed by exacerbated levels of IFN-γ (Moroda et al., 2017). Additionally, although contributing to the development of Th17 response during *T. gondii* infection (Passos et al., 2010), high levels of the cytokine IL-6 can be detrimental to tissue integrity (Rochet et al., 2015).

In addition to the systemic inflammation observed during infection, the presence of marked inflammatory infiltrate in the lung environment was evidenced by the higher number of leukocytes, especially CD4^+^ and CD8^+^ T lymphocytes, in the BALF of mice infected by both strains (ME49 or CK4). Mice infected with the CK4 strain exhibited a significantly higher number of CM and EM CD4^+^ T lymphocytes, in addition to CD8^+^ T cells during acute infection, and these findings were sustained in the early chronic stage as well. Our results, and other similar findings show that different immune system components are differently activated in infection with atypical strains when compared to conventional typical strains (Bernstein et al., 2020; Brito et al., 2023b).

Histopathological analysis revealed a greater impairment of the pulmonary architecture in animals infected with the atypical strain CK4, during both acute and chronic stages, along with a larger mononuclear cell infiltrate. Additionally, inflammatory cytokines IFN-γ, IL-12, IL-6 and TNF-α, as well as nitric oxide, were found elevated in the BALF of infected mice, highlighting the ability of *T. gondii* to induce airway inflammation. The increased tissue damage in the lungs caused by CK4 infection, compared to ME49, may be related to the persistence of activated macrophages capable of producing pro-inflammatory cytokines (Park and Hunter, 2020), contributing to greater tissue damage (Montoya and Liesenfeld, 2004; Melchor and Ewald, 2019), and by disrupting anion channels in the airway epithelial cells and contributing to airway inflammation (Qiu et al., 2023).

The sustained airway inflammation induced by *T. gondii* infection can be associated with increased levels of total proteins observed in the BALF, as higher cellular damage leads to a higher accumulation of proteins in the lungs, which could be indicative of pulmonary alveolar proteinosis (PAP), characterized by the accumulation of lipoprotein material in the alveolar spaces due to impairment of resident cells and inflammatory infiltrate (Kelly and McCarthy, 2020). As presented here, the inflammatory pattern observed in the airways of *T. gondii-*infected mice may be a contributing factor to pulmonary dysregulation, resulting in protein accumulation, tissue damage and the potential development of PAP. The alveolar wall is covered by a layer of pulmonary surfactant, mainly composed of phospholipids, which not only prevents alveolar collapse during breathing but is also important in defence against external agents. Dysfunction in the pulmonary environment, such as architectural compromise, can disrupt the concentration of these proteins, leading to PAP (Suzuki and Trapnell, 2016). In this sense, more studies are still necessary to analyse the impact of *T. gondii* infection in the lung homeostasis and how it could contribute to the accumulation of surfactant and other components in the lower respiratory tract.

The findings presented here highlights significant differences in the course of *T. gondii* infection induced by CK4 strain compared to ME49 infection, raising pertinent questions regarding infection with atypical *T. gondii* strain. Our study provides valuable insights into the immunopathogenesis of infection induced by an atypical strain, shedding light on the potential differences between atypical and classical strains of *T. gondii*. These observations open up ways for further research and deeper exploration of the dynamics and impact of atypical strains in the context of *T. gondii* infection.

## Supporting information

Bastilho et al. supplementary material Bastilho et al. supplementary material

## Supplementary material

The supplementary material for this article can be found at https://doi.org/10.1017/S0031182025000538.
