# Supplementary figures and images for: Toxoplasma gondii non-archetypal strain induces lung inflammation during acute and early chronic infection in mice
Source: Parasitology. 2025 Apr 14;152(5):497–509. doi: 10.1017/S0031182025000538 (PMC12278009; doi:10.1017/S0031182025000538)

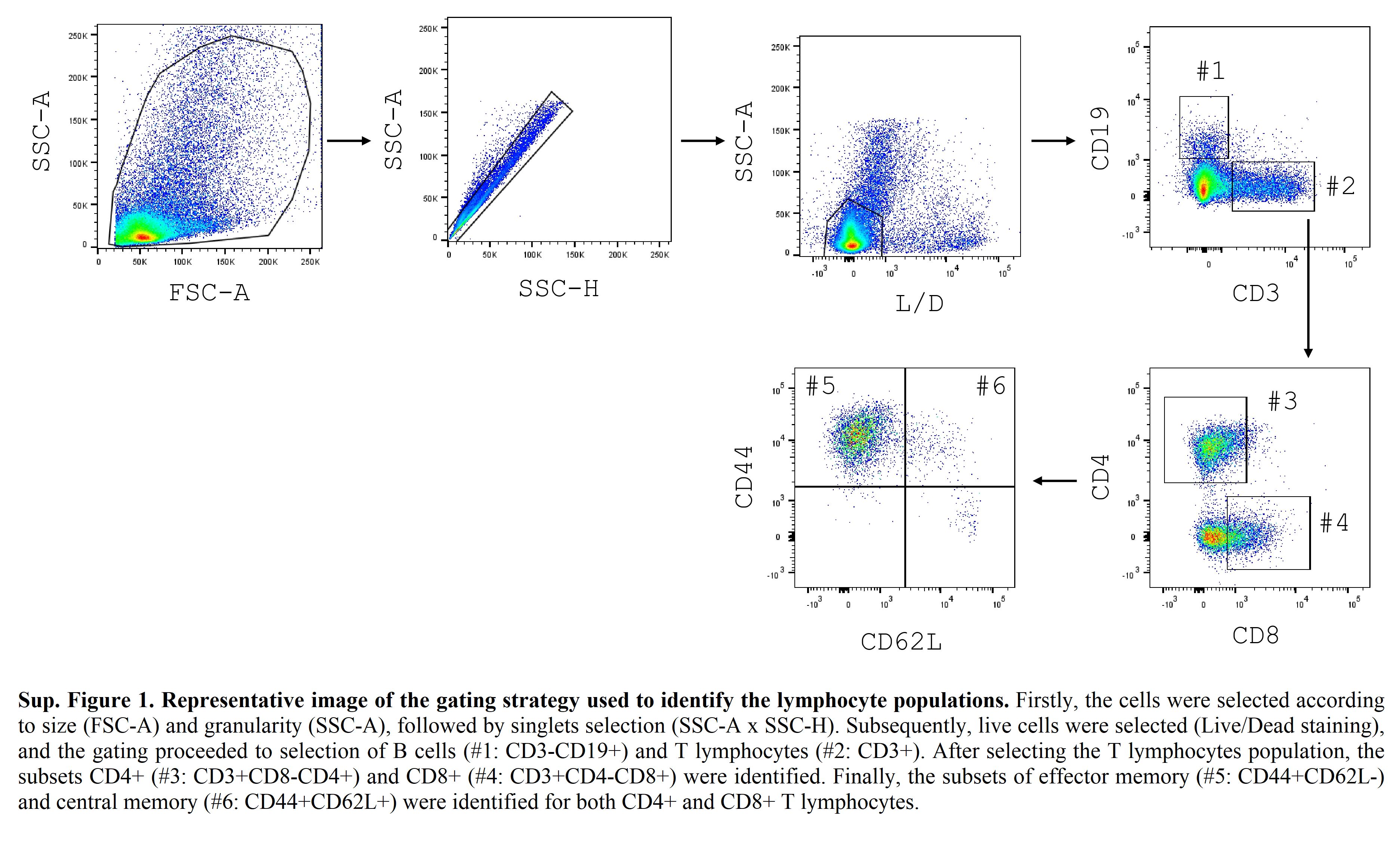

Supplement: Bastilho et al. supplementary material [file S0031182025000538sup001.tif]
